# Hyperexcitability and impaired intracortical inhibition in patients with fragile-X syndrome

**DOI:** 10.1038/s41398-019-0650-z

**Published:** 2019-11-20

**Authors:** Florence Morin-Parent, Camille Champigny, Angelina Lacroix, François Corbin, Jean-François Lepage

**Affiliations:** 1 Department of Pediatrics, Faculty of Medicine and Health Sciences, Sherbrooke University, Sherbrooke, Canada; 2 Sherbrooke University Hospital Research Center, Sherbrooke, Canada; 3 Department of Biochemistry, Faculty of Medicine and Health Sciences, Sherbrooke University, Sherbrooke, Canada

**Keywords:** Autism spectrum disorders, Physiology

## Abstract

Fragile-X syndrome (FXS) is characterized by neurological and psychiatric problems symptomatic of cortical hyperexcitability. Recent animal studies identified deficient γ-aminobutyricacid (GABA) inhibition as a key mechanism for hyperexcitability in FXS, but the GABA system remains largely unexplored in humans with the disorder. The primary objective of this study was to assess GABA-mediated inhibition and its relationship with hyperexcitability in patients with FXS. Transcranial magnetic stimulation (TMS) was used to assess cortical and corticospinal inhibitory and excitatory mechanisms in 18 patients with a molecular diagnosis of FXS and 18 healthy controls. GABA-mediated inhibition was measured with short-interval intracortical inhibition (GABA_A_), long-interval intracortical inhibition (GABA_B_), and the corticospinal silent period (GABA_A+B_). Net intracortical facilitation involving glutamate was assessed with intracortical facilitation, and corticospinal excitability was measured with the resting motor threshold. Results showed that FXS patients had significantly reduced short-interval intracortical inhibition, increased long-interval intracortical inhibition, and increased intracortical facilitation compared to healthy controls. In the FXS group, reduced short-interval intracortical inhibition was associated with heightened intracortical facilitation. Taken together, these results suggest that reduced GABA_A_ inhibition is a plausible mechanism underlying cortical hyperexcitability in patients with FXS. These findings closely match those observed in animal models, supporting the translational validity of these markers for clinical research.

## Introduction

Fragile-X syndrome (FXS) is a rare disorder, but the leading monogenic cause of autism spectrum disorder and the first hereditary cause of intellectual disability. FXS results from the methylation of the Fmr1 gene, which leads to a marked reduction or absence of the fragile-X mental retardation protein (FMRP), an important regulator of protein synthesis involved in brain development and synaptic function^1^. While the clinical phenotype may vary considerably between individuals affected by the disorder, it typically involves psychiatric and neurological manifestations indicative of neuronal hyperexcitability, including seizures, anxiety, hyperactivity, hypersensitivity, and hyperarousal^2,3^. Accumulating EEG evidence supports the existence of cortical hyperexcitability in patients with FXS^4–7^. However, the physiological mechanisms involved in hyperexcitability in FXS remain poorly understood. Research in this field is of prime clinical importance since correcting the alterations involved in neuronal and circuit excitability could alleviate several core symptoms of the disorder^2^.

Hyperexcitability is a consistent observation across animal models of the disorder^3,8,9^, and is believed to result from an imbalance between excitatory and inhibitory drives in intracortical circuits^3,10^. The dominant view over the past 15 years has been that the absence of FMRP in FXS induces an overactivation of metabotropic glutamate receptors (mGluRs), thereby increasing neuronal excitability^11^. Although experimental data from Fmr1-ko animals support the idea that mGluRs play a role in the process^12,13^, clinical trials using mGlurR antagonist for FXS have been unsuccessful^14,15^. Moreover, there is evidence suggesting that mGluR overactivity alone is insufficient to fully explain hyperexcitability in FXS^16–18^. In that regard, several recent studies show the presence of dysfunctions affecting diverse elements of the GABAergic system in various animal models of FXS^17–21^, suggesting that reduced inhibition is a key mechanism for circuit hyperexcitability. This theory is further supported by in vivo evidence confirming that defective inhibition from GABAergic interneurons is causally involved in the behavioral and sensory phenotype of the fly and mouse FXS models^17,18^. These findings suggest that the GABAergic system may be a promising therapeutic target to correct circuit hyperexcitability in FXS^2^.

The GABA system remains surprisingly unexplored in humans with FXS, despite several past and ongoing clinical trials aiming to modulate it^22,23^ (NCT03697161; NCT01911455). To date, the most significant evidence of a GABA dysfunction in humans with FXS comes from Hulst and colleagues (2015), who showed diminished GABA_A_ receptor binding throughout the brain in a small group of patients using positron-emission tomography (PET) (*n* = 10). Yet, it is unknown if this reduction in receptor availability translates into functional alterations at the cortical level. Transcranial magnetic stimulation (TMS) is valuable tool to answer this question, as this technique is capable of probing the excitatory and inhibitory mechanisms in the cortex of awake humans^24–26^. TMS is a painless and non-invasive neurostimulation technique that uses magnetic fields to stimulate a restricted part of the brain. TMS acts on the cortex through the recruitment of interneurons in upper cortical layers mediating GABAergic inhibition onto pyramidal neurons^27^, whose resulting activity closely match what is seen non-invasively and at a macroscopic level in humans following stimulation of the primary motor cortex^28^. Abundant evidence demonstrates the sensitivity of TMS to GABAergic mechanisms in healthy and clinical populations^24,25,29,30^. Because the technique is safe, well tolerated, and does not require extensive training or sedation to be used with people with intellectual disability, TMS is well suited for the study of the GABA system in FXS.

In the present study, we used TMS to investigate intracortical inhibition and excitation in a cohort of individuals with a molecular diagnosis of FXS (*n* = 18). We assessed three measures of GABAergic inhibition, including short-interval intracortical inhibition (SICI; GABA_A_ mediated), long-interval intracortical inhibition (LICI, GABA_B_ mediated), and the corticospinal silent period (CSP; GABA_A+B_). Resting motor threshold (rMT) was used to assess corticospinal excitability, and net intracortical excitation involving glutamate was measured with intracortical facilitation (ICF). We hypothesized that GABA-mediated inhibition would be reduced, while corticospinal excitability and ICF would be enhanced in individuals with FXS compared to healthy controls.

## Material and methods

### Participants

Individuals aged 13–50-year old with a molecular diagnosis of FXS were eligible to participate in the study. Age and sex-matched control participants were eligible to take part in the study if they reported being in good general health, exempt of genetic or chronic conditions, with no history of neurological, neurodevelopmental, or psychiatric disorders. All control participants were alcohol, caffeine, and medication free at the moment of testing. Exclusion criteria comprised: any absolute contraindication for TMS, untreated hypothyroidism, being pregnant, or breastfeeding. Additional exclusion criteria for FXS patients comprised: change in medication in the previous 3 months, being diagnosed with epilepsy, taking more than three psychoactive drugs, and being unable to comply to simple verbal commands.

Twenty patients with a molecular diagnosis of FXS (two females), and 20 age and sex-matched healthy controls were recruited. Data from one male FXS patient were rejected due to technical EMG recording issues, and multivariate outliers were identified and removed using the Mahalanobis distance (*p* < 0.05), resulting in 18 patients in each group (one FXS, two controls, all males). Power analysis showed that this sample size allowed the detection of an effect size of *f*  = 0.5 (large effect) in 83% of the cases with an alpha level of 0.05 (two-tails). Seven FXS patients were taking psychoactive medication, four of them taking two types of medications or more (Tables 1 and 2); none of them had a diagnosis of epilepsy. The legal guardian of each participant provided signed informed consent. The study was approved by the research ethics board from the Sherbrooke Hospital Research Center and conducted in accordance with the 1964 Declaration of Helsinki.

**Table 1:** Sample characteristics.

Characteristics	FXS *N* = 18	Controls *N* = 18
Age in years (mean, interquartile range)	24.88 (7.5)	23.88 (5.3)
IQ (mean, interquartile range)	49.64 (17.5)	–
Intellectual disability (*n*, %)	18 (100%)	–
Sex		
Male	16 (89%)	16 (89%)
Female	2 (11%)	2 (11%)
Mutation (*n*, %)		
Full	16 (89%)	–
Mosaic	2 (11%)	–
Medication status (*n*, %)		
None	11 (61.1%)	18 (100%)
One	3 (16.6%)	–
Two or more	4 (22.2%)	–

*IQ* Intellectual quotient

**Table 2:** Sex, age, and medication at time of testing for FXS patients.

Patient	Sex, age y	Psychoactive medication mg/d
FXS 1	M, 20	Abilify 2, Olanzapine 2.5, Zoloft 50
FXS 2	M, 40	None
FXS 3	M, 26	None
FXS 4	M, 18	None
FXS 5	F, 18	None
FXS 6	M, 35	Venlafaxine 75,
FXS 7	M, 28	Quetiapine 50, Risperidone 2, Sertraline 50
FXS 8	M, 21	None
FXS 9	M, 26	Sertraline 50
FXS 10	M, 26	None
FXS 11	M, 20	None
FXS 12	M, 27	None
FXS 13	M, 14	Abilify 2, Adderal XR 40
FXS 14	M, 26	Citalopram 40
FXS 15	M, 25	None
FXS 16	M, 24	Sertraline 25, Quetiapine 25, Clonidine 0.1
FXS 17	F, 19	None
FXS 18	M, 35	None

### TMS procedure

TMS was performed with a Magstim BiStim-2 using a 70 mm figure-of-eight coil (Magstim Compagny Limited, UK). To identify the optimal stimulation site, the coil was initially positioned over the C3 location of the International 10–20 EEG system at an intensity of 35% of maximum stimulator output (MSO). Single pulse stimulations were administered at a 1 cm interval covering a 16 cm^2^ region centered at C3, and the process was repeated while increasing the intensity (5% MSO steps) until a motor-evoked potential was obtained in the contralateral first dorsal interosseus (FDI) muscle. The location was then entered in the neuronavigation system (BrainSight, Rogue Research, Canada), and stimulations of decreasing intensity (2% MSO steps) were administered in the vicinity (±1 cm) to identify the most sensitive location. Electromyographic signals (EMG) were recorded with surface electrodes, amplified using a Powerlab 4/30 system, digitized at 4 KHz, and recorded with Scope v4.0 for offline analysis.

**Fig. 1:**
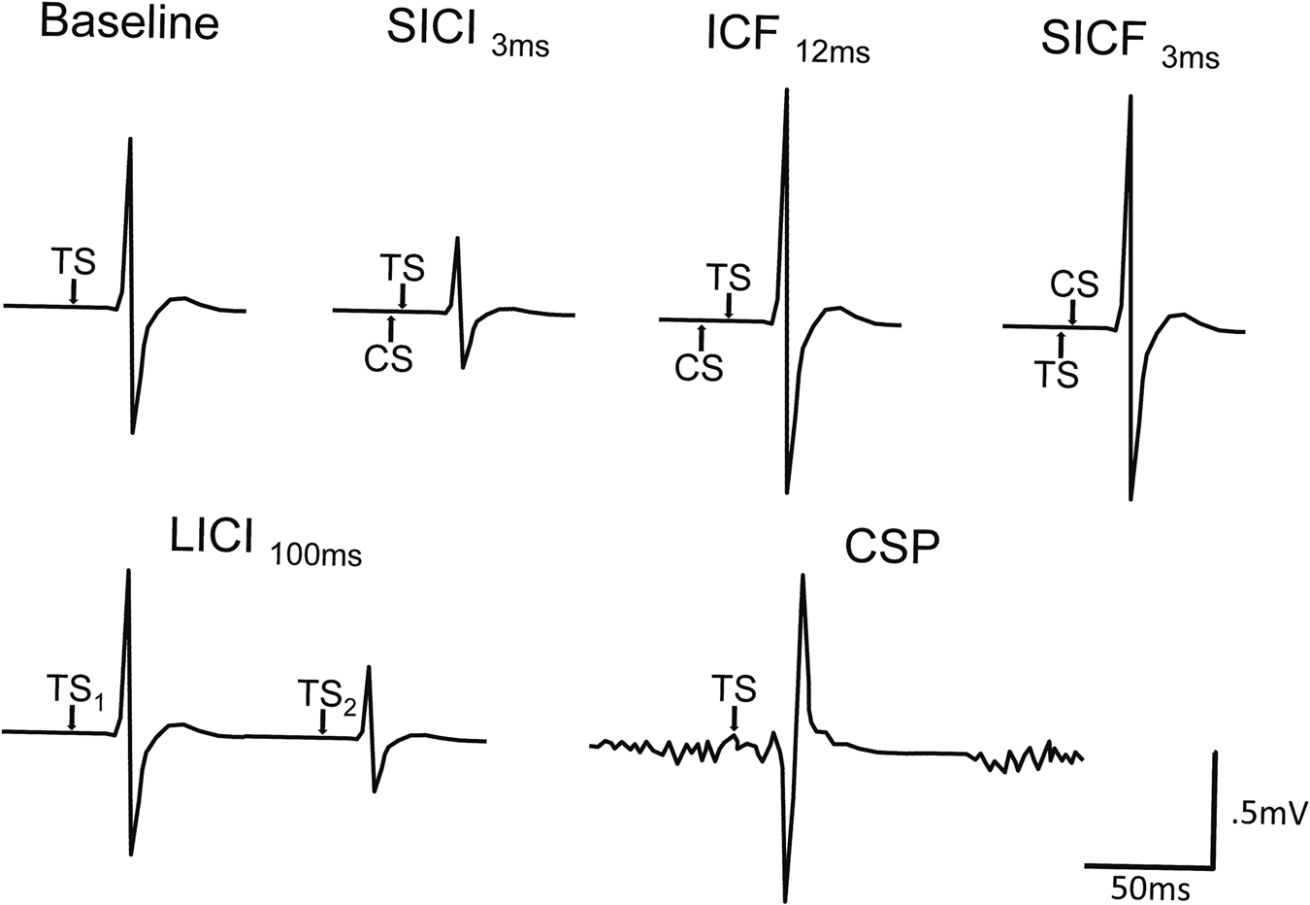
Typical EMG responses to the TMS stimulation protocols. For SICI, ICF, and SICF, the average peak-to-peak MEP amplitude resulting from the CS-TS combination is compared to the baseline response induced by the TS alone in order to compute the degree of inhibition or facilitation. For LICI, the MEP amplitude induced by the second TS is compared to the amplitude of the MEP evoked by the first TS. The CSP is the duration between the onset of the MEP to the return of baseline EMG activity. For paired-pulse measures, the interval between TMS pulses is specified in milliseconds (ms). CS conditioning stimulus, CSP corticospinal silent period, ICF intracortical facilitation, LICI long-interval intracortical inhibition, SICI short-interval intracortical inhibition, SICF short-interval intracortical facilitation, TS test stimulus.

Standard TMS procedures were used^31^ and administered following the guidelines from the International Federation of Clinical Neurophysiology^32^. Briefly, resting motor threshold (rMT) was established as the minimal intensity to induce MEPs of at least 50 µV amplitude in five out of ten consecutive trials using the relative frequency criterion^33^, and intracortical facilitation (ICF, 12 ms interpulse interval (IPI)) was acquired to measure net intracortical excitability. Tests for intracortical inhibitory functions included short-interval intracortical inhibition (SICI; 3 ms IPI; GABA_A_) and long-interval intracortical inhibition (LICI, 100 ms IPI; GABA_B_). Corticospinal silent period (CSP; GABA_A+B_) was acquired while participants performed an isometric voluntary contraction of the FDI corresponding to 20% of maximal muscular contraction. In a subset of participants (*n* = 20; 10 FXS), short-interval intracortical facilitation (SICF) was acquired using the same interval as SICI (3 ms IPI) to assess the potential contribution of I-wave facilitatory components on SICI^34^. Conditioning stimuli (CS) for SICI, ICF, and the test stimulus for SICF, were set to an intensity corresponding to 80% of rMT; baseline MEPs and all other stimulations were administered using a test stimulus (TS) intensity set to induce MEPs of 1 mV peak-to-peak amplitude (≈125% of rMT). Fifteen trials were acquired for each measure, except for CSP (*n* = 5). A frameless neuronavigation system (BrainSight, Rogue Research) was used to ensure stable coil positioning during testing. The procedure was well tolerated by all participants and there was no serious adverse event. Typical EMG responses for each TMS protocol are depicted in Fig. 1.

### Data processing and statistical analysis

Individual EMG epochs were visually inspected and rejected if visible muscular activity was present in the 100 ms time window preceding the TMS pulse. Peak-to-peak MEP amplitude was calculated and averaged for baseline, SICI, ICF, and SICF independently. Ratios were then computed for SICI, ICF, and SICF, using the mean baseline MEP as the denominator. For LICI, the amplitude of the MEP evoked by the TS was divided by the one induced by the CS within each trial, and the resulting ratios were averaged^35^. CSP was measured independently by two raters as the duration from the MEP onset to the return to baseline EMG activity (inter rater agreement, *r* = 0.950). For each measure, a minimum of 10 valid trials were kept, resulting in Cronbach’s alpha being superior to 0.90^36^. Between-group differences were assessed using independent samples two-tailed t-tests (adjusted for unequal variance when Levene’s test *p* < 0.05) and multiple comparisons were handled with false-discovery rate (adjusted *q*-values). Data are available from the authors upon request.

## Results

**Fig. 2:**
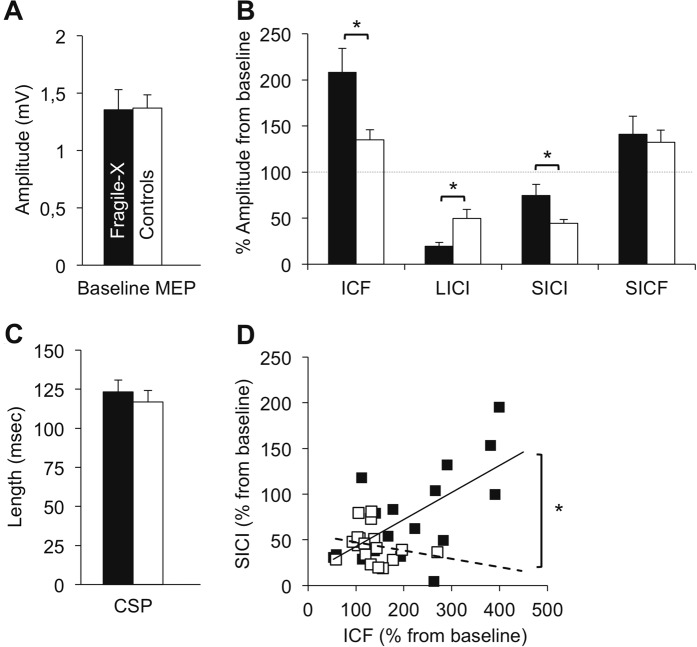
TMS results. **a** Amplitude of baseline motor-evoked potentials (MEP) for each group (in mV). **b** Corticospinal silent period duration (CSP) duration; **c** Results of paired-pulse stimulation protocols expressed in ratio from baseline. Significant differences were found on intracortical facilitation (ICF), short-intracortical inhibition (SICI), and long-interval cortical inhibition (LICI). Short-interval cortical facilitation (SICF) did not differ between groups. **d** Association between SICI and ICF in each group. A significant positive correlation was present in the FXS group, and significantly different from the one in the control group. Error bars indicate standard error of the mean.

FXS patients and controls did not differ regarding rMT (*T*_32.4_ = 1.97, *q* = 0.43), amplitude of baseline MEP (*T*_29.4_ = 0.68, *q* = 0.44), or CSP length (*T*_33_ = 0.77, *q* = 0.43) (Fig. 2a, b). However, FXS patients showed increased ICF (*T*_22.8_ = 2.60, *q* = 0.017), increased LICI (*T*_22.7_ = 2.90, *q* = 0.011), and reduced SICI (*T*_21.7_ 2.41, *q* = 0.021) compared with controls. Groups did not differ significantly on SICF (*T*_18_ = 0.37, *q* = 0.43) (Fig. 2c). Since deficient GABA_A_ inhibition has been associated with circuit hyperexcitability in animal models of FXS^17,37^, we explored the relationship between SICI and ICF. Correlation analysis showed that FXS patients with less SICI had larger ICF (*r* = 0.648, *q* = 0.011), indicating a positive relationship between intracortical hypo-inhibition and hyperexcitability. This relationship was significantly different from the one observed in the control group (*r* = −0.222 in controls; Fisher’s *z* = 2.73; *q* = 0.011) (Fig. 2d). Because TMS measures are sensitive to several pharmacological agents^29^, we ran exploratory analyses (non-corrected) comparing only non-medicated FXS with healthy controls. Results corroborated observations made at the whole-group level, with non-medicated patients differing significantly from controls on SICI (*T*_26_ = 3.71, *p* = 0.001) and ICF (*T*26 = 2.38, *p* = 0.025), and LICI showing a similar trend (*T*_26_ = 1.96, *p* = 0.060). The correlation between SICI and ICF remained significant in the non-medicated FXS group (*r* = 0.737; *p* = 0.010), and significantly different from the control group (Fisher’s *z* = 2.67; *p* = 0.007). Comparing medicated FXS patients with non-medicated FXS patients and controls did not show significant differences on any TMS measures (Mann–Whitney–Wilcoxon tests), possibly due to the small number of medicated FXS patients (*n* = 7). Excluding FXS females from the analysis did not change the overall pattern of results. LICI and ICF remained significant (*T*_32_ > 2.31, <0.05), the relationship between SICI and ICF remained significant in the FXS group (*r* = 0.635, *p* = 0.008), and significantly different from the control group (Fisher’s *z* = 2.57, *p* = 0.010). The most notable difference was that the *p* value for SICI was now slightly above the significance threshold (*T*_32_ = 1.98, *p* = 0.057).

## Discussion

Recent evidence from animal studies showed the pivotal role of GABAergic inhibitory interneurons in the neurophysiological phenotype of FXS^17,18,37^. However, it remained unclear how these abnormalities translated to the human brain. The current study bridges this gap by providing evidence that GABA_A_-mediated intracortical inhibition is reduced in humans with FXS, and that this alteration co-occurs with increased intracortical circuit excitability.

It is recognized that SICI reflects the activity of cortical interneurons on post-synaptic GABA_A_ receptor^29^. Importantly, since groups did not differ on SICF, it is unlikely that between-group differences in SICI level result from a contamination by indirect facilitatory waves^34^. The presence of altered GABA_A_ mediated inhibition concurs with the only previous study directly assessing GABAergic function in FXS patients, where [11C]-flumazenil PET was used to show a reduction in GABA_A_ receptor binding^38^. These results are in line with preclinical evidence showing a reduction of GABA synthesizing enzyme and GABA_A_ receptor mRNA levels in animal models of the disorder^2^. Similarly, the number of parvalbumin expressing interneurons, the most abundant type of GABAergic interneurons in the cortex^39,40^, is reduced in Fmr1 KO animals^41^. Considering the fact that TMS is believed to interact predominantly with parvalbumin interneurons^42,43^, a reduction in this cell population could also contribute to the aberrant intracortical inhibition observed in FXS patients. Interestingly, recent evidence suggests a causal role between parvalbumin dysfunctions and impairments in sensory processing in FXS, a behavioral phenotype that is common to both animals and humans^18^. Sensory processing was not evaluated in the present study, thus the association between TMS measures of inhibition and the magnitude of sensory abnormalities in FXS patients remains to be investigated.

The between-group difference observed on ICF confirms the presence of circuit hyperexcitability in FXS patients, as was previously inferred by EEG studies^4,5,7,44^. Because ICF reflects, at least in part, the activity of NMDA receptors^45^, one explanation for this observation is that the increase in ICF is directly linked to the exacerbation of glutamate signaling described in animal models of the disorder^3,11^. However, considering that it is also modulated by other neurotransmitter systems, ICF can more accurately be conceived as “glutamatergic facilitation tempered by GABAergic inhibition”^46^, namely because the time interval at which ICF can be observed (6–20 ms) encompasses the tail of the GABA_A_-mediated inhibition measured by SICI^47^. Hence, while it is established that ICF and SICI depend on distinct neuronal populations, it is their interaction that ultimately controls the corticospinal output^29,48^. Further supporting a relationship between GABA_A_-mediated inhibition and intracortical excitability, pharmacological manipulations that reduce or enhance SICI have the opposite effect on ICF (reviewed in^29^), and changes in SICI can mediate variations in ICF^49^. Here, the fact that reduced SICI is associated with increased circuit excitability in FXS patients, as expressed by ICF, concurs with recent evidence obtained from in vivo animals pointing to faulty GABA_A_ inhibition as a primary cause for circuit hyperexcitablity in the absence of FMRP^17,18^. Although the present data cannot ascertain that the alteration in GABA_A_ signaling is a causative factor of hyperexcitability in FXS, it nonetheless supports the significance of the GABA_A_ system as a potential target to correct a core neurophysiological feature of FXS.

Surprisingly, FXS patients showed enhanced LICI, which is primarily mediated through the activity of post-synaptic GABA_B_ receptors^50–52^. The increase in LICI may explain why CSP values did not differ between both groups despite reduced SICI, as CSP amalgamates both GABA_A_ and GABA_B_ inhibition^29^. Although the mechanism responsible for the increase in LICI observed in FXS patients is elusive, it could reflect a compensatory mechanism. Indeed, there is cross talk between GABA_A_ and GABA_B_ receptors at the post-synaptic site in such a way that activation of GABA_B_ receptors increases GABA_A_ inhibition^53^. Whether such cross talk is at play in FXS remains to be established. However, the preservation of LICI can be related to evidence from mice models showing that post-synaptic GABA_B_ receptors are spared in FXS, in contrast with what is observed at the presynaptic level^19,20^. Specifically, reduced presynaptic GABA_B_ signaling has been reported at glutamatergic synapses^19^, while excessive presynaptic GABA_B_ signaling appears to be present at inhibitory synapses^20^. Since presynaptic GABA_B_ receptors are involved in auto-inhibition and regulate neurotransmitter release, both alterations are susceptible to contribute to circuit hyperexcitability. The presence of increased GABA_B_ mediated inhibition described here, coupled with the apparent heterogeneity in terms of GABA_B_ receptor function may explain, at least in part, the limited efficacy of selective GABA_B_ agonists for treating FXS in recent clinical trials^22,23^.

**Fig. 3:**
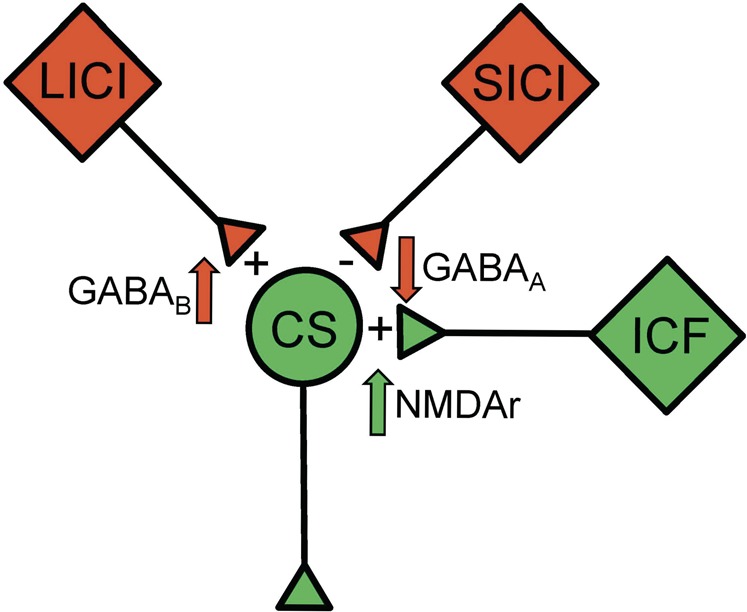
Schematic representation of the main findings. Inhibitory circuits are in red and excitatory circuits in green. The “plus” and “minus” signs indicate the observed effect compared to controls, and the nearby arrows specify the presumed mechanisms involved. CS corticospinal neuron, ICF intracortical facilitation, NMDAr *N*-methyl-d-aspartate receptors, LICI long-interval intracortical inhibition, SICI short-interval intracortical inhibition.

The lack of difference in rMT between controls and FXS patients is intriguing considering that FMRP influences the activity of ion channels^54^, including voltage gated sodium channels which are believed to play a role in the cortical hyperexcitability of FXS^55,56^. It is however known that rMT is normal in several disorders associated with cortical hyperexcitability^30,57^, including in patients with Dravet syndrome, which is caused by a mutation of a gene directly involved in the proper function of voltage gated sodium channels^24^. It is possible that variables unrelated to neuronal function, such as the scalp-to-cortex distance^58^ and cerebral tissue structure^59,60^, may have obscured potential between-group differences. These evidence, coupled with abundant TMS data from epilepsy patients^30^, suggest that the rMT is less sensitive to hyperexcitability than paired-pulses protocols. Figure 3 recapitulates the main findings and the presumed underlying neurotransmitter dysfunctions of FXS.

Interestingly, the overall pattern of TMS abnormalities of FXS patients is strikingly similar to what is seen in patients with a missense mutation of the γ2 subunit of the GABA_A_ receptor (GABRG2(R43Q)), which is associated with an hereditary form of generalized epilepsy^57^. These patients exhibit normal rMT and CSP, but display reduced SICI, and an increase in ICF of similar magnitude to the one observed in FXS patients^57^. Furthermore, patients with a missense mutation of the γ2 subunit show reduced [11C]-flumazenil binding^61^, like FXS patients^38^. Given that [11C]-flumazenil interacts with benzodiazepine binding sites, it is possible that the reduction in binding observed in FXS patients does not result from an under expression of the γ2 subunit per se, but from the diminished expression of other GABA_A_ receptor subunits composing this binding site, including α1,3, β1,2 and γ2 subunits, which are all under expressed in animal models of FXS^62,63^. It thus remains to be seen if the same alterations are responsible for the reduction in SICI and [11C]-flumazenil binding in FXS. It is also noteworthy that the TMS alterations common to FXS and GABRG2(R43Q) patients differ markedly from those seen in disorders involving other aspects of the GABAergic system, including patients with succinic semialdehyde dehydrogenase (SSADH) deficiency^25^, who lack an enzyme involved in GABA metabolism, and Prader-Willi syndrome, caused by deletion or imprinting defect of genes encoding α5, β3, and γ3 subunits of GABA_A_ receptors^64^. Considering that all of these disorders show a ubiquitous reduction of [11C]-flumazenil binding^38,61,65,66^, the sensitivity of TMS to specific elements of the GABAergic system emerges as being particularly enlightening for clinical research in neurogenetic disorders associated with inhibitory dysfunctions.

The lack of objective outcome measures and biomarkers sensitive to the mechanisms targeted by experimental drugs is a major hurdle for clinical trials in FXS^67–69^. Biomarkers would benefit clinical trials by allowing a stratification of the clinical population and providing non-biased outcome measures. Eventually, they could be used to predict response to treatment and design personalized therapies. Because the consequences of FXS are primarily cognitive, emotional, and behavioral, there is tremendous interest in objectively assessing the impact of interventions on brain function. In that regards, interesting advances have been made with EEG^70^. Namely, auditory evoked EEG responses appeared partially normalized in FXS children treated with minocycline^71^, and recent data showed remarkably similar electrocortical responses in terms of neural synchronization between FXS patients and Fmr1 KO mice^72,73^, an important step towards the implementation of translational-relevant biomarkers in clinical trials. While additional research is required to assess the translational value of TMS markers for FXS, the upcoming results from two ongoing clinical trials including TMS as a secondary outcome could provide some answers (NCT02680379; NCT03722290). The unique features of TMS provide an interesting complement to the widely available EEG, notably by allowing for the discrimination of which inhibitory and excitatory processes aggregated in the EEG response are being modulated by an intervention. This is particularly relevant considering that all EEG markers put forth for FXS are hypothesized to involve, directly or indirectly, faulty GABAergic inhibition as an underlying mechanism^6,71,73^.

### Limitations

Although large for a study involving FXS patients, the sample size remains limited, which might have induced type II errors. The heterogeneity of the sample in terms of medication status can also be considered as a limitation. However, more than half of our sample was medication free and still displayed alterations identical to those observed at the whole-group level, showing that our results were not due to the presence of psychoactive drugs. Due to time constraint and to maximize collaboration from low functioning patients, relevant measures such as input-output recruitment curves and late cortical disinhibition^74^, could not be performed. As it is usually the case in clinical studies with TMS, measures were obtained from stimulation of the primary motor cortex. It thus remains to be seen whether the alterations reported here extend to other cortical areas. This might well be the case, as the decrease in GABA_A_ receptor binding reported by Hulst and colleagues^38^ seems fairly homogenous across the brain of FXS patients. The use of simultaneous TMS-EEG protocols could help assess the functional impact of this decrease in regions beyond the primary motor cortex. Indeed, using EEG to measure the brain response to TMS stimulations, recent studies have shown that paired-pulse TMS applied to the dorsolateral prefrontal cortex of healthy individuals induces EEG evoked potentials that are consistent with the electrophysiological responses typically derived from MEPs using SICI, ICF, and LICI protocols^75,76^. Although technically challenging, this approach could provide valuable insight on the inhibition-excitation imbalance across the cerebral cortex of patients with FXS.

## Conclusion

This study shows the presence of aberrant inhibitory mechanisms in patients with FXS. These alterations offer a plausible explanation for the cortical hyperexcitability typical of this disorder, and suggest new avenues for pharmacological interventions targeting the GABAergic system. Further research is required to elucidate the precise neurochemical mechanisms responsible for the physiological alterations reported here. While the GABAergic system holds promises as a therapeutic target, the increase in GABA_B_ inhibition illustrates the complex dynamics that are likely to be at play in terms of inhibitory mechanisms in FXS. These findings illustrate the usefulness of TMS in assessing intracortical excitability in FXS and, possibly, in monitoring it following treatment. These results also highlight the crucial need to better characterize the neurophysiology of humans with the disorder in order to accelerate the discovery of new treatment avenues.
